# Heme-Induced ROS in Trypanosoma Cruzi Activates CaMKII-Like That Triggers Epimastigote Proliferation. One Helpful Effect of ROS

**DOI:** 10.1371/journal.pone.0025935

**Published:** 2011-10-11

**Authors:** Natália Pereira de Almeida Nogueira, Cintia Fernandes de Souza, Francis Monique de Souza Saraiva, Pedro Elias Sultano, Sergio Ranto Dalmau, Roberta Eitler Bruno, Renata de Lima Sales Gonçalves, Gustavo Augusto Travassos Laranja, Luís Henrique Monteiro Leal, Marsen Garcia Pinto Coelho, Claudio A. Masuda, Marcus F. Oliveira, Marcia Cristina Paes

**Affiliations:** 1 Laboratório de Interação Tripanossomatídeos e Vetores, Departamento de Bioquímica, Instituto de Biologia Roberto Alcântara Gomes (IBRAG), Universidade do Estado do Rio de Janeiro (UERJ), Rio de Janeiro, Brasil; 2 Laboratório de Microscopia e Processamento de Imagens, Universidade do Estado do Rio de Janeiro (UERJ), Rio de Janeiro, Brasil; 3 Laboratório de Artrópodos Hematófagos, Instituto de Bioquímica Médica, Universidade Federal do Rio de Janeiro (UFRJ), Rio de Janeiro, Brasil; 4 Laboratório de Bioquímica Redox - Instituto de Bioquímica Médica, Universidade Federal do Rio de Janeiro (UFRJ), Rio de Janeiro, Brasil; 5 Laboratório de Biologia Molecular de Leveduras, Programa de Biologia Molecular e Biotecnologia, Instituto de Bioquímica Médica, Universidade Federal do Rio de Janeiro (UFRJ), Rio de Janeiro, Brasil; 6 Laboratório de Inflamação e Metabolismo, Instituto Nacional de Ciência e Tecnologia de Biologia Estrutural e Bioimagem (INBEB), Universidade Federal do Rio de Janeiro (UFRJ), Rio de Janeiro, Brasil; 7 Instituto Nacional de Ciência e Tecnologia - Entomologia Molecular (INCT-EM), Universidade Federal do Rio de Janeiro (UFRJ), Rio de Janeiro, Brasil; Agency for Science, Technology and Research - Singapore Immunology Network, Singapore

## Abstract

Heme is a ubiquitous molecule that has a number of physiological roles. The toxic effects of this molecule have been demonstrated in various models, based on both its pro-oxidant nature and through a detergent mechanism. It is estimated that about 10 mM of heme is released during blood digestion in the blood-sucking bug's midgut. The parasite *Trypanosoma cruzi*, the agent of Chagas' disease, proliferates in the midgut of the insect vector; however, heme metabolism in trypanosomatids remains to be elucidated. Here we provide a mechanistic explanation for the proliferative effects of heme on trypanosomatids. Heme, but not other porphyrins, induced *T. cruzi* proliferation, and this phenomenon was accompanied by a marked increase in reactive oxygen species (ROS) formation in epimastigotes when monitored by ROS-sensitive fluorescent probes. Heme-induced ROS production was time-and concentration-dependent. In addition, lipid peroxidation and the formation of 4-hydroxy-2-nonenal (4-HNE) adducts with parasite proteins were increased in epimastigotes in the presence of heme. Conversely, the antioxidants urate and GSH reversed the heme-induced ROS. Urate also decreased parasite proliferation. Among several protein kinase inhibitors tested only specific inhibitors of CaMKII, KN93 and Myr-AIP, were able to abolish heme-induced ROS formation in epimastigotes leading to parasite growth impairment. Taken together, these data provide new insight into *T. cruzi*- insect vector interactions: heme, a molecule from the blood digestion, triggers epimastigote proliferation through a redox-sensitive signalling mechanism.

## Introduction

*Trypanosoma cruzi*, the etiologic agent of Chagas disease [1], during its life cycle, develops and differentiates within the midgut lumen of triatomine insects. The transmission of parasites to vertebrate hosts occurs through the insect's faeces when the triatomine vectors feed on blood, which usually comprises an intake of approximately 6 to 12 times its own body weigth. The amount of blood ingested is equivalent to about 10 mM of heme, which is present in different forms inside the triatomine digestive tract [2]. The absence of a complete heme biosynthetic pathway in both *T. cruzi* and *Leishmania* support the hypothesis that heme is essential for the survival of these parasites [3]–[5]. Thus, it seems plausible that trypanosomatids should acquire extracellular heme from their invertebrate hosts. In this regard, our group has previously demonstrated that heme stimulated *T. cruzi* epimastigote proliferation in a dose-dependent manner [6].

Ferriprotoporphyrin-IX (heme) constitutes a key molecule in many biological reactions, including respiration, detoxification and oxygen transport [7], processes that are essentially mediated by heme proteins such as cytochromes, catalase, myoglobin and hemoglobin. However, *“free”* heme exerts a number of toxic effects, causing not only molecular damage to lipids, DNA and proteins [8]–[10], but also decomposing organic hydroperoxides into highly reactive alkoxyl and peroxyl radicals that are included in the pool of reactive oxygen species (ROS) [11]–[12]. In addition, due to its amphiphilic features, heme can associate with lipid membranes, leading to altered membrane permeabilisation and cell disruption markedly a redox independent mechanism [13].

It is known that cells are capable of generating endogenously and constitutively ROS which are utilized in the induction and maintenance of signal transduction pathways involved in cell growth and differentiation [14]. However, a high level of pro-oxidant species overcomes the cells pro-oxidant/antioxidant balance disturbing the redox signalling and control [15]–[18]. An aberration in endogenous ROS production, known as oxidative stress, has been involved in the process of aging [16] and in the pathogenesis of several diseases such as cancer [19], diabetes [20] and atherosclerosis [21]. Conversely, the regulated increase in free radicals leads to a temporary imbalance that represents the physiological basis for redox regulation [22]. Several cytokines [23], growth factors [24] and hormones [25] trigger ROS production. In response to these triggers, ROS act as secondary messengers in the intracellular signal transduction pathway in normal physiological events [14], [26], [27].

A multifunctional serine/threonine protein kinase that responds to changes in the redox state of cells is the Ca^2+^ calmodulin kinase II (CaMKII) [28], [29]. CaMKII is known to mediate the downstream effects of Ca^2+/^ CaM [30]. CaMK II holoenzyme contains three main regions: an N-terminal catalytic region responsible for catalyzing the phosphotransferase reaction, a regulatory region that contains Ca^2+/^CaM binding sites and an auto inhibitory domain (AID). In the absence of bound Ca^2+^/CaM, the CaMKII is maintained in an inactive state because of an interaction of the AID with the catalytic domain of its own subunit [30]. The Ca^2+^/CaM complex binding induces the phosphorylation of the CaMKII in Thr^286^ and enhancing its kinase activity [30].

We have recently shown that heme-induced *T. cruzi* growth is associated with calcium-calmodulin-dependent kinase II (CaMKII) activity [31]. Based on previous evidence showing that heme can exert potent pro-oxidant actions [8], [11] and that CaMKII activity can be stimulated by oxidation [28], [29], here we hypothesized whether heme would drive *T. cruzi* proliferation through a redox dependent CaM Kinase II-like cascade and in fact, the data presented herein indicate that heme induces a transient oxidative stress condition that stimulates *T. cruzi* growth via a mechanism mediated by a CaM Kinase II-like pathway.

## Materials and Methods

### Chemicals

Rabbit anti-α/β tubulin polyclonal antibody was purchased from Sigma-Adrich Fine Chemicals (St. Louis, MO, USA). Hemin and other porphyrins were from Frontier Scientific (Logan, UT, USA). 5-(and-6)-chloromethyl-2′,7′dichlorodihydrofluorescein diacetate acetyl ester (CMH_2_-DCFDA) and Dihydroethidium (DHE) were purchased from Invitrogen Corporation (Carlsbad, California, USA). Mouse monoclonal anti-4-hydroxy-2-nonenal (4-HNE) antibody was from Abcam Inc. (Cambridge, UK). Anti-mouse secondary antibody was from GE Healthcare (Uppsala, Sweden). The inhibitors used in the work were from Calbiochem (La Jolla, CA, USA). All other reagents used were of analytical purity.. .

### Parasites

*Trypanosoma cruzi* Dm28c (CT-IOC-010) strain was provided by the Trypanosomatid Collection of the Oswaldo Cruz Institute, Fiocruz, Brazil. Parasites were grown at 28 °C for 7 days in brain–heart infusion medium (BHI) and supplemented with 30 µM hemin (heme-Cl) and 10% foetal calf serum (FCS). Parasite growth was monitored by cell counting in a Neubauer chamber. Unless otherwise indicated, the parasites were adapted for two passages in BHI supplemented with 10% FCS without heme supplementation prior to the experiments.

### Effects of porphyrins on *T. cruzi* proliferation

Epimastigotes stocks were maintained in BHI supplemented with 10% FCS and 30 µM heme. For the experiments,Cells were harvested from culture the flasks, washed twice in BHI and suspended in fresh BHI, 10% FCS without the addition of heme. Next, 2.5×10^6^ parasites/mL were grown at 28 °C for 10 or 12 days in BHI medium supplemented with 10% FCS in the absence or presence of different concentrations of porphyrins. Parasite proliferation was monitored by cell counting in a Neubauer chamber.

### Effect of urate on *T. cruzi* proliferation

Epimastigotes were maintained in BHI supplemented with 10% FCS and 30 µM heme for 7 days. Next, 2.5×10^6^ parasites/mL were grown at 28 °C for 10 days in BHI medium supplemented with 10% FCS in the absence or presence of 30 µM heme and 1 mM urate. Parasite growth was monitored by cell counting in a Neubauer chamber.

### Effects of H_2_O_2_ and CaMKII inhibition on *T. cruzi* proliferation

Epimastigotes were maintained in BHI supplemented with 10% FCS and 30 µM heme for 7 days. Next, 2.5×10^6^ parasites/mL were grown at 28 ° in BHI medium supplemented with 10% FCS in the absence (control) or in the presence of 20 µM H_2_O_2_, 30 µM heme, 30 µM Myr-AIP, 30 µM Myr-AIP plus 20 µM H_2_O_2_ and 30 µM Myr-AIP plus 30 µM heme. Parasite proliferation was monitored by cell counting in a Neubauer chamber after 5 days of culture

### Fluorescence microscopy

Parasites were collected by centrifugation at 1500 *g* for 5 min (Hermle-z323k, rotor: 220.72V04) and washed in PBS (100 mM phosphate buffer and 150 mM NaCl, pH 7.4). The cells (1×10^7^) were re-suspended in PBS and incubated with 2 µM CM-H_2_DCFDA and different concentrations of heme at 28 °C for 30 min. Aliquots of cells were mounted on slides and coverslips and were observed by differential interference contrast (DIC) and fluorescence using an Axioplan 2 Zeiss fluorescence microscope (Zeiss, Göttingen, Germany). All obtained images were processed equivalently with Adobe PhotoShop software (Adobe, Seattle, USA).

Flow cytometry - Epimastigotes (1×10^7^cells/mL) were loaded in PBS (100 mM phosphate buffer and 150 mM NaCl, pH 7.4) with 2 µM CM-H_2_DCFDA or 5 µM DHE for 30 min and 20 µM H_2_O_2_, 30 µM heme or other porphyrins for 15 min unless otherwise stated. ROS production was analysed by flow cytometry using a FACS Calibur apparatus with a 488 nm ion-argon laser (BD Biosciences, Mississauga, Canada). Controls with classical antioxidants were carried out by pre-incubating parasites with 1 mM urate for 15 min or 5 mM GSH for 2 h prior to heme exposure. To analyse the effect of CaMKII inhibition, parasites were pre-incubated with 2 µM KN-93 or 30 µM Myr-AIP for 1 h.

### Hydrogen peroxide production

For the H_2_O_2_ release measurements, adapted epimastigotes (1×10^7^cells/mL) were loaded in PBS (100 mM phosphate buffer and 150 mM NaCl, pH 7.4) containing 1.25 µM of Amplex Red reagent and 1 U/mL horseradish peroxidise and 30 µM heme for 30 min. Afterwards, parasites were separated by centrifugation at 2000 *g* for 5 min and then the supernatants were analysed in a Cary Eclipse fluorescence spectrophotometer using an excitation wavelength of 530 nm and an emission wavelength of 590 nm. Data were calibrated by adding increasing concentrations of a freshly prepared H_2_O_2_ solution.

### Thiobarbituric acid reactive substances (TBARS)

Adapted epimastigotes were collected by centrifugation at 1500 *g* for 5 min and washed twice in PBS (100 mM phosphate buffer and 150 mM NaCl, pH 7.4). The pellet was re-suspended in the reaction solution (PBS containing 200 µM desferoxamin and 2 mM CaCl_2_) and incubated in the absence or presence of different concentrations of heme and 300 µM H_2_O_2_ at 37 °C for 30 min. The cells were then lysed by freezing and thawing, and the cell-free extracts of *T. cruzi* were incubated at 95 °C for 30 min with 2% trichloroacetic acid (TCA) and 0.134% thiobarbituric acid (TBA). Next, the tubes were cooled, and 500 µL of n-butanol was added. Thiobarbituric acid reactive substances (TBARS) were separated by centrifugation at 9300 *g* for 5 min, the supernatants was collected and the absorbance was measured at 532 nm.

### Western blotting

Adapted epimastigotes (4×10^8^cells/mL) were incubated in BHI supplemented with 10% FCS in the absence (control) or in the presence of 30 µM heme at 28 °C f or 30 minutes. Parasites were collected by centrifugation at 1500 *g* for 5 min, washed with PBS and the pellet was re-suspended in lysis buffer (50 mM HEPES, 1 mM MgCl_2_, 1% Triton X-100, 0.1% SDS, 10 mM EDTA; pH 6.4).containing protease inhibitor cocktail Sigma-Adrich Fine Chemicals (1.04 mM AEBSF, 800 nM aprotinin, 20 µM leupeptin, 40 µM bestatin, 15 µM pepstatin A and 14 µM E-64). After parasite lysis, the samples were centrifuged at 9300 *g*, 4 °C for 10 min, the pellet was discarded and the supernatants were used for protein quantification according to Lowry *et al*. [32]. Whole protein extracts of *T. cruzi* (about 80 µg) were subjected to electrophoresis in a 15% SDS-polyacrylamide gel under reducing conditions. Proteins were transferred to a nitrocellulose membrane at 4 °C for 2 h. Membranes were blocked with Tris-buffered saline (25 mM Tris, 192 mM glicine, pH 8.3, 20% methanol) solution containing 0.1% Tween 20 (TBS-T) and 5% bovine serum albumin (BSA). The membranes were then incubated overnight with anti-4-HNE antibody (1∶1000), or anti-α/β tublin (1∶1000) diluted in blocking solution, washed in TBS-T, and finally incubated for 1 h with horseradish peroxidase-conjugated anti-mouse antibody or horseradish peroxidase-conjugated anti-rabbit antibody (1∶10.000). The bands were revealed by chemiluminescence using the ECL substrate. Blots were exposed to ECL Hyperfilm (Amersham) and quantification was performed by densitometric analysis of the exposed films (Adobe Photoshop 5 programme) using anti- α/β tublin as a load control.

### Effects of kinase inhibitors on *T. cruzi*

In the experiments employing protein kinase inhibitors, the drugs were initially used at the following final concentrations, corresponding to five-fold the *Ki* values for mammalian cells: LY294002 (phosphatidylinositol 3-kinase inhibitor, 8.0 µM); roscovitine (inhibitor of cyclin-dependent kinases; 3.5 µM); H-89 (inhibitor of cyclic AMP-dependent protein kinase, 0.24 µM); H-9 (inhibitor of cyclic GMP-dependent protein kinase 5 µM); bisindolylmaleimide I (inhibitor of protein kinase C, 0.05 µM); KN-93 (inhibitor of CaM kinase II, 2 µM); Myr-AIP (inhibitor of CaM kinase II, 30 µM).

### Statistical analyses

Statistical analysis were conducted with GraphPad Prism 3 software (GraphPad Software, Inc., San Diego, CA). Data are presented as the mean ± standard deviation (SD), and all experiments were repeated at least three times. Data were analysed by one-way analysis of variance (ANOVA), and differences between groups were assessed with Tukey's post-test. The level of significance was set at *p*<0.05.

## Results

### *T. cruzi* proliferation is induced specifically by heme but not by other porphyrins

**Figure 1:**
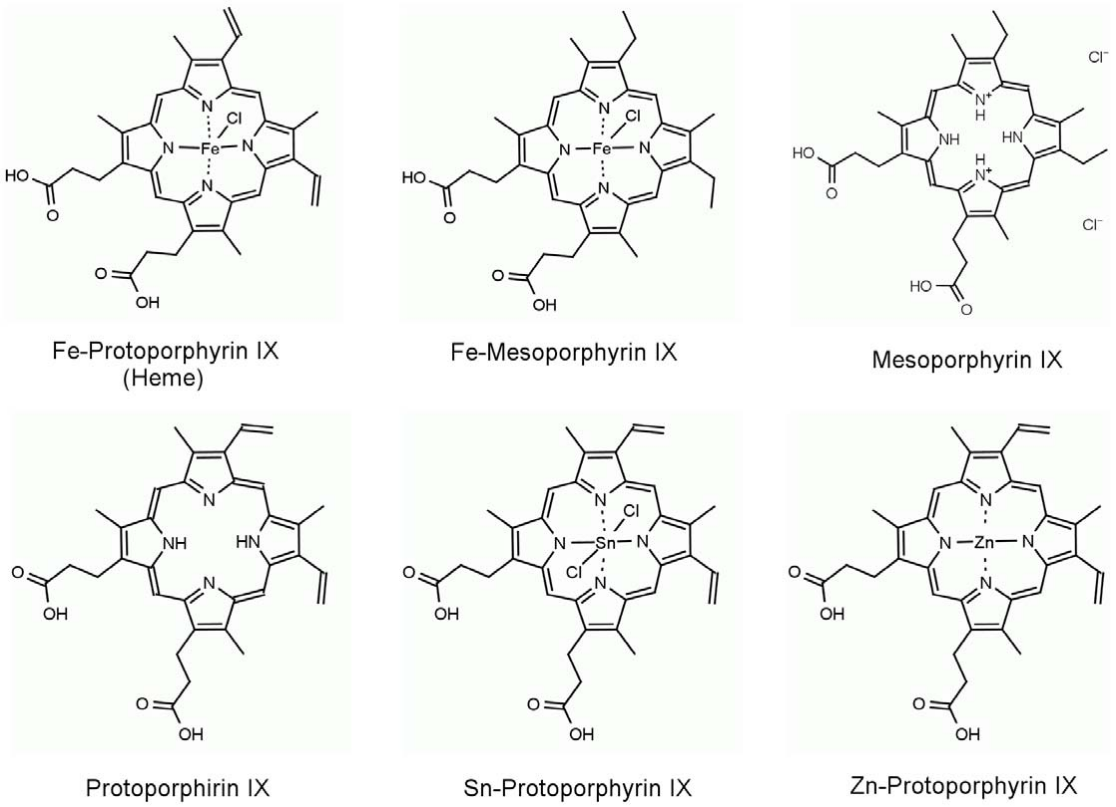
Molecular structure of the different porphyrins utilised in this study.

**Figure 2:**
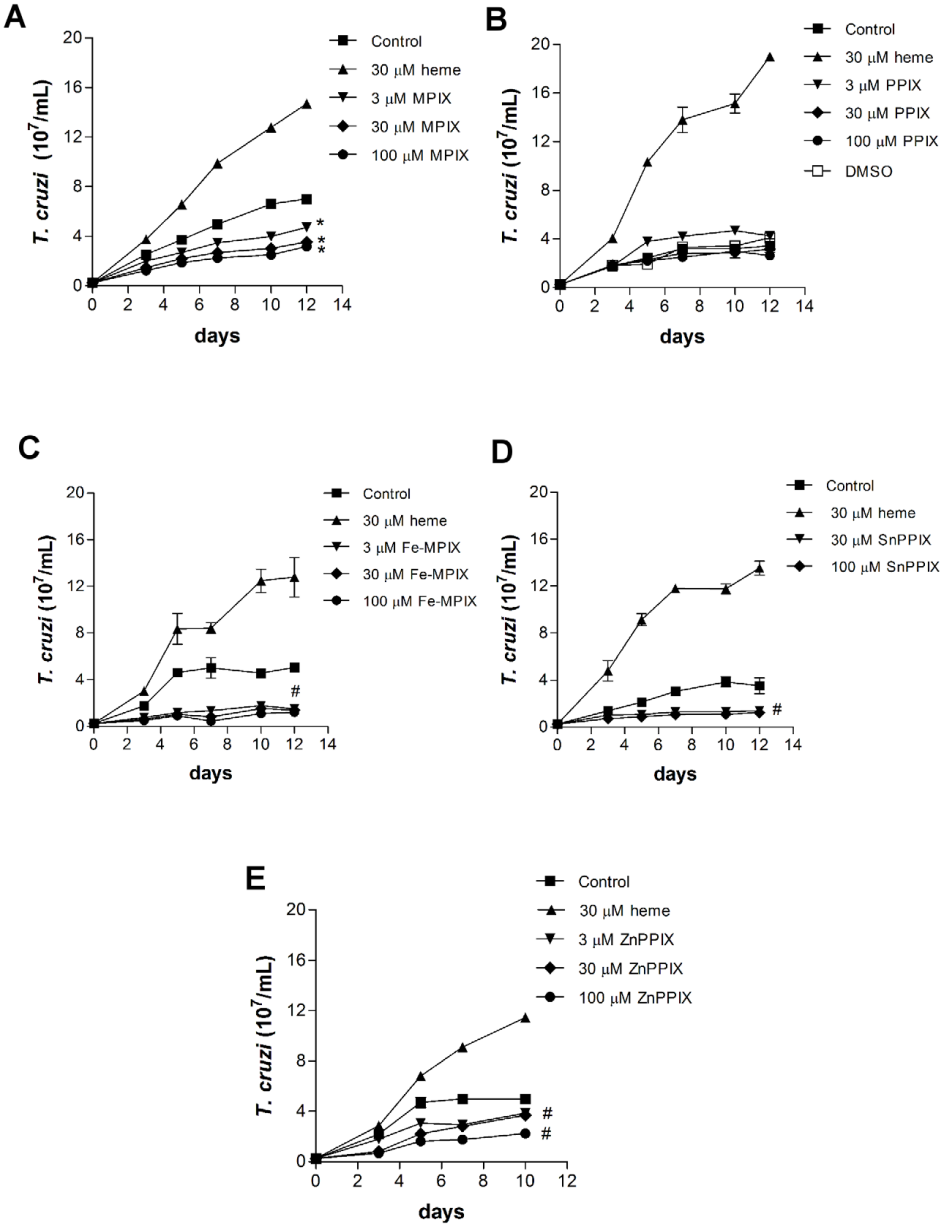
Porphyrins and *T. cruzi* proliferation. Epimastigotes (2.5×10^6^ cells/mL) were incubated in BHI medium supplemented with 10% FCS in the absence (control) or in the presence of the porphirins (**A**) mesoporphyrin IX (MPIX), (**B**) protoporphyrin IX (PPIX), (**C**) Fe-mesoporphyrin IX (Fe-MPIX), (**D**) Sn-protoporphyrin IX (SnPPIX), (**E**) Zn-protoporphyrin IX (ZnPPIX), for ten or twelve days. All data are presented as the mean ± standard deviation (n = 3), * *p*<0.001 or # p<0.05 as compared to the control group by Tukey's test.

Previous data from our group have shown that heme, but not hemoglobin or its peptides, stimulates *T. cruzi* proliferation *in vitro* in a dose-dependent manner [6]. Thus, to investigate the structural determinant of the heme molecule that causes *T. cruzi* proliferation, cells were treated with several heme analogues (Figure 1). We tested porphyrins that lack the central iron atom: protoporphyrin IX (PPIX) and mesoporphyrin IX (MPIX). We also used Fe- mesoporphyrin IX (Fe-MPIX), which has a structure similar to heme but lacks the two vinyl groups, which are replaced by two ethyl groups, as well as MPIX. Others porphyrins such as SnPPIX and ZnPPIX were also used. Figure 2 shows that among all of the porphyrins tested, only heme was able to induce a potent proliferative effect on *T. cruzi*. In fact, treatment with MPIX, Fe-MPIX and ZnPPIX for 10 or 12 days significantly impaired *T. cruzi* proliferation even at low concentrations (3 µM). We also added free iron to cell cultures and the proliferation was not increased (data not show). Thus, these results show that the ferriprotoporphyrin (heme) molecule, and not other porphyrins, is required to potentiate *T. cruzi* growth. Interestingly, the vinyl groups are also important for heme-induced *T. cruzi* growth, indicating that the central iron is not solely responsible for the observed effects.

### Heme, but not other porphyrins, induces reactive species (ROS) formation in *T. cruzi*

**Figure 3:**
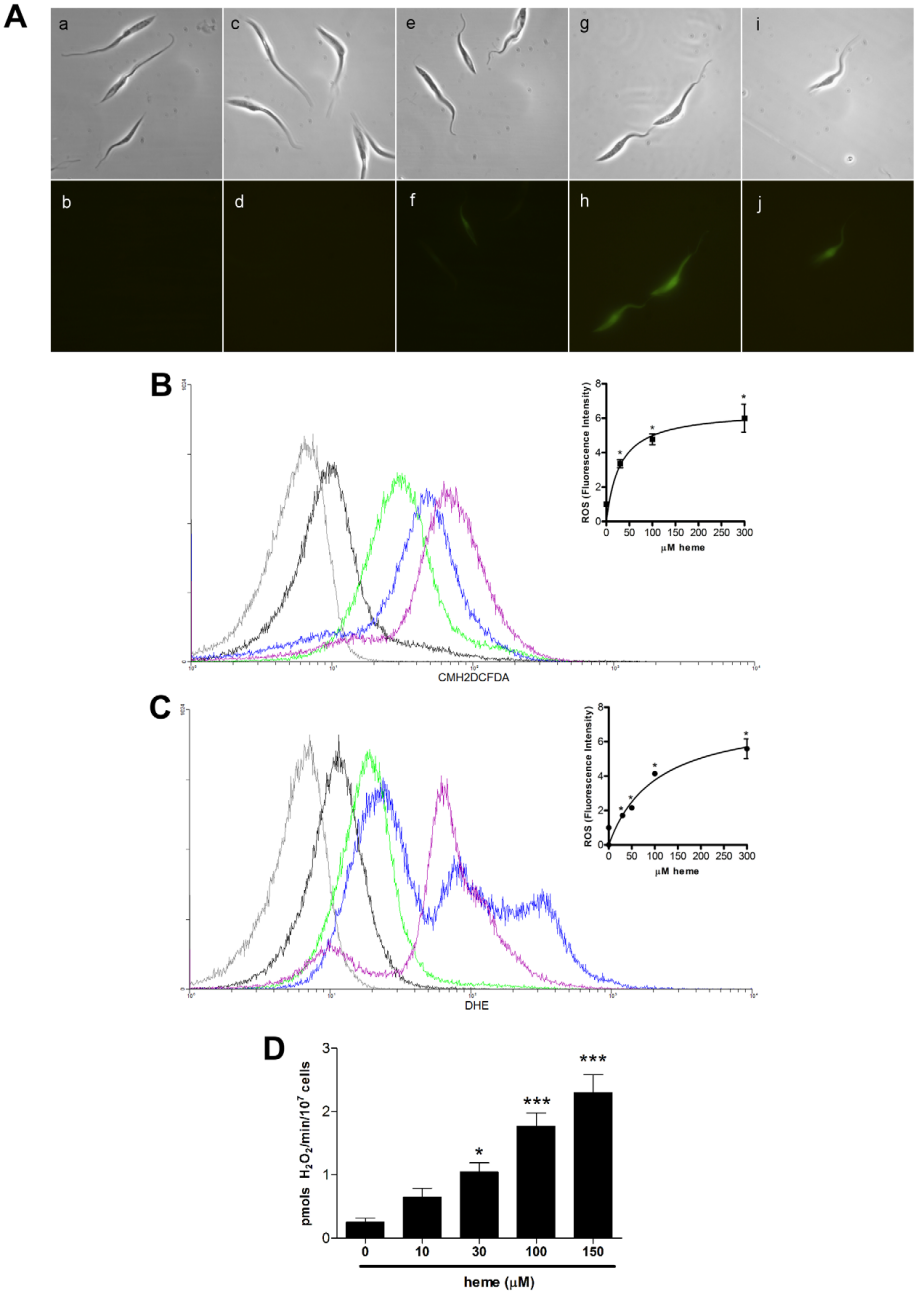
Effect of heme on ROS formation in *T. cruzi* epimastigotes. (**A**) Epimastigotes (1×10^7^ cells/mL) were incubated with 2 µM CMH_2_DCFDA and different concentrations of heme. Images a, c, e,g and i show differential interference contrast, whereas b, d, f, h and j show fluorescence images. The CMH_2_DCFDA (2 µM) signal indicated in green was acquired with λEm 517–527 nm: (a and b) autofluorescence, (c and d) no heme, (e and f) 30 µM heme, (g and h) 100 µM heme and (i and j) 300 µM heme. (**B**) Epimastigotes (1×10^7^cells/mL) were incubated in PBS with 2 µM CMH_2_DCFDA and 30 µM heme for 30 min. The ROS formation was analysed by flow cytometry. The histograms correspond to: autofluorescence (gray), 2 µM CMH_2_DCFDA (control-black), 30 µM heme (green), 100 µM heme (blue) and 300 µM heme (purple). The histograms are representative of five independent experiments. The inset graph represents the fluorescence intensity values obtained by the ratio of the experimental group median to the control group median (without heme). Data are expressed as the mean ± standard deviation (n = 5), * *p*<0.001 as compared to the control group by Tukey's test. (**C**) Epimastigotes (1×10^7^cells/mL) were incubated in PBS with 5 µM DHE and heme for 30 min, and ROS formation was measured by flow cytometry. The histograms show autofluorescence (gray), 5 µM DHE (control-black), 30 µM heme (green), 100 µM heme (blue) and 300 µM heme (purple). The histograms are representative of two independent experiments. The inset graph represents the fluorescence intensity values obtained by the ratio of the experimental group median to the control group median (without heme). Data are expressed as the mean ± standard deviation (n = 2), * *p*<0.001 as compared to the control group by Tukey's test. (**D**) Epimastigotes (1×10^7^cells/mL) were incubated in PBS with 1.25 µM Amplex red, 1 U/mL HRP and 30 µM heme for 30 min, and H_2_O_2_ production was measured in the supernatant by fluorescence spectrophotometry. Data are expressed as the mean ± standard deviation (n = 3), * *p*<0.01, ** *p*<0.05 and *** * *p*<0.001 as compared to the control group by Tukey's test.

**Figure 4:**
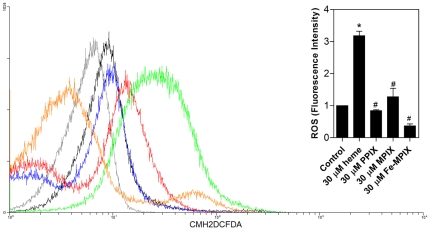
Porphyrins and ROS formation *T. cruzi*. Epimastigotes (1×10^7^ cells/mL) were incubated in PBS with 2 µM CMH_2_DCFDA for 30 min with the addition of 30 µM heme, 30 µM PPIX, 30 µM MPIX or 30 µM Fe-MPIX for the final 15 min. The production of ROS was analysed by flow cytometry. The histograms show autofluorescence (gray), 2 µM CMH_2_DCFDA (control-black), 30 µM heme (green), 30 µM PPIX (blue), 30 µM MPIX (red) and 30 µM Fe-MPIX (orange). The histograms are representative of four independent experiments. The inset graph represents the fluorescence intensity values obtained by the ratio of experimental group median to the control group median (without heme). Data are expressed as the mean ± standard deviation (n = 2), * *p*<0.001 as compared to the control group and ^#^ *p*<0.001 relative to the heme group by Tukey's test.

It has been known that heme is able to promote peroxides and others reactive species formation [11] but in the case of *T. cruzi*, heme induces proliferation as well Thus, to gain insight on the mechanism by which heme promotes *T. cruzi* proliferation, we next investigated ROS formation in heme-exposed *T. cruzi* by measuring the fluorescence intensities of two distinct ROS-sensitive probes: CMH_2_-DCFDA and dihydroethidium (DHE). Figure 3A shows that heme caused a dose-dependent increase of ROS formation in *T. cruzi*, as assessed by the CMH_2_-DCFDA fluorescence signal using an epifluorescence microscope. Heme treatment did not affect parasite viability or structure because they appeared to be well preserved and to possess the expected, normal shape (Figure 3A, DIC images). We then evaluated ROS formation in parasites treated with several heme concentrations by measuring the CMH_2_-DCFDA fluorescence signal using flow cytometry (Figure 3B). There was a clear dose-dependent increase in CMH_2_-DCFDA fluorescence in the parasites treated with increased heme concentrations (Figure 3B). Heme-induced ROS production was further investigated with another ROS-sensitive probe, DHE. Figure 3C show that, similarly to the data obtained for CMH_2_-DCFDA, heme caused a dose-dependent increase in DHE fluorescence intensity. Additionally, we employed Amplex red reagent in combination with horseradish peroxide (HRP), to detect H_2_O_2_ released from heme treated epimastigotes. As shown in figure 3D H_2_O_2_ levels increased in a dose-response manner to the addition of heme. To check if the heme analogues are also able to promote the ROS formation in parasites, we measured the CMH_2_-DCFDA fluorescent signal using flow cytometry (Figure 4). Despite of the structural similarities with heme, the challenge of epimastigotes with 30 µM PPIX, 30 µM MPIX or 30 µM Fe-MPIX was unable to induce ROS, confirming that heme, is in fact, required to trigger ROS formation in *T. cruzi*.

### Heme induces lipid peroxidation in *T. cruzi*

**Figure 5:**
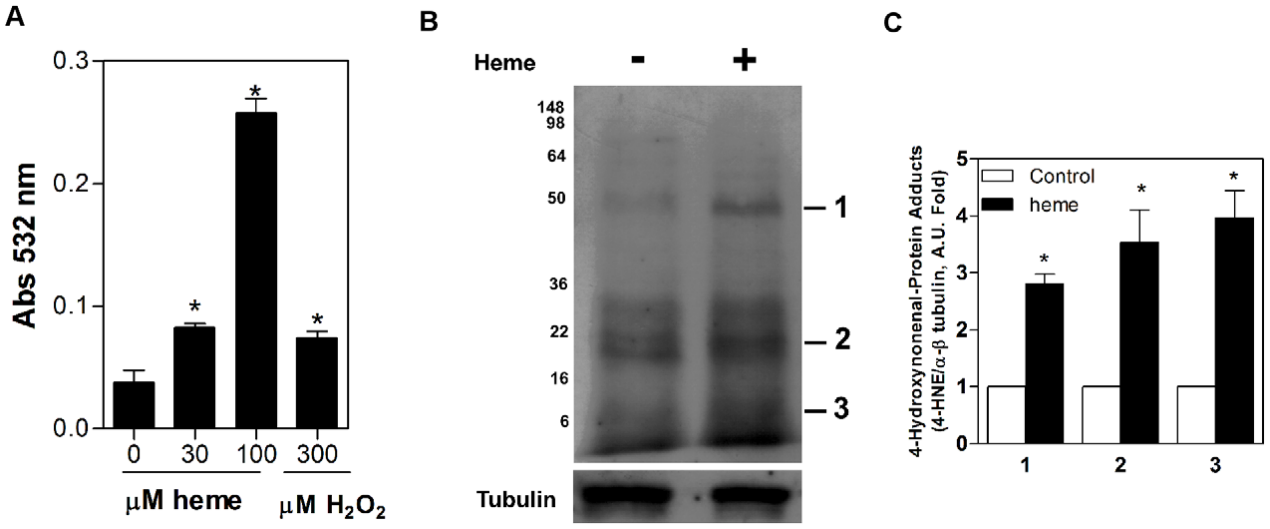
Heme triggers lipid peroxidation in *T. cruzi*. (**A**) Epimastigotes (1×10^9^ cells /mL) were challenged with 30 µM, 100 µM heme or 300 µM H_2_O_2_ (as a positive control) at 37 °C for 30 min. Next, the cells were lysed, and the cell-free extracts were incubated at 95 °C for 30 min in acidic TBA. Lipid peroxides were extracted in *n*-butanol and separated by centrifugation. The supernatant was used to determine the absorbance at 532 nm. Data are expressed as the mean ± standard deviation (n = 3), * *p*<0.05 as compared to the control group (no heme) by Tukey's test. (**B**) Epimastigotes (4×10^8^ cells /mL) were incubated in BHI supplemented with 10%FCS, in the absence (control without heme) or in the presence of 30 µM heme for, 30 min. Parasites were lysed, and 80 µg of whole protein were electrophoresed in a 15% PAGE gel, transferred onto a nitrocellulose membrane, and incubated with monoclonal anti-4-HNE (1∶1000). The bands were visualised using the ECL kit. This result is representative of three independent experiments. (**C**) Quantification of the level of 4-HNE adducts was determined by densitometry of three independent experiments using α/β tubulin as a load control. Data are expressed as the mean ± standard deviation (n = 3), * *p*<0.001 as compared to the control group (without heme) by Tukey's test. The bands were analyzed using Adobe Photoshop 5.0.

Lipid peroxidation is one of the hallmarks of the pro-oxidant effects of heme [8]. Lipid peroxides are usually decomposed into reactive aldehydes such as malondialdehyde (MDA) and 4-hydroxy-2-nonenal (4-HNE), which are also reactive oxygen species [33]. Figure 5 shows that both end-products of lipid peroxidation were observed in cellular extracts of *Trypanosoma cruzi*, and their levels were increased in the presence of heme. In fact, heme induced a significant, dose-dependent increase in MDA formation (Figure 5A). Due to its electrophilic properties, the aldehyde 4-HNE forms adducts with cellular proteins [26], [33]–[35]. In Figure 5B, we can observe a great increment in 4-HNE-protein adduct formation after heme exposure. The α/β tubulin antibody was used to normalize the protein load. The protein oxidation seems to target specific proteins, named as bands 1–3, which increased 4-HNE label in about 3 to 4 fold when compared to the control group (without heme) (Figure 5C).

### Antioxidants prevent heme-induced ROS and impair *T. cruzi* proliferation

**Figure 6:**
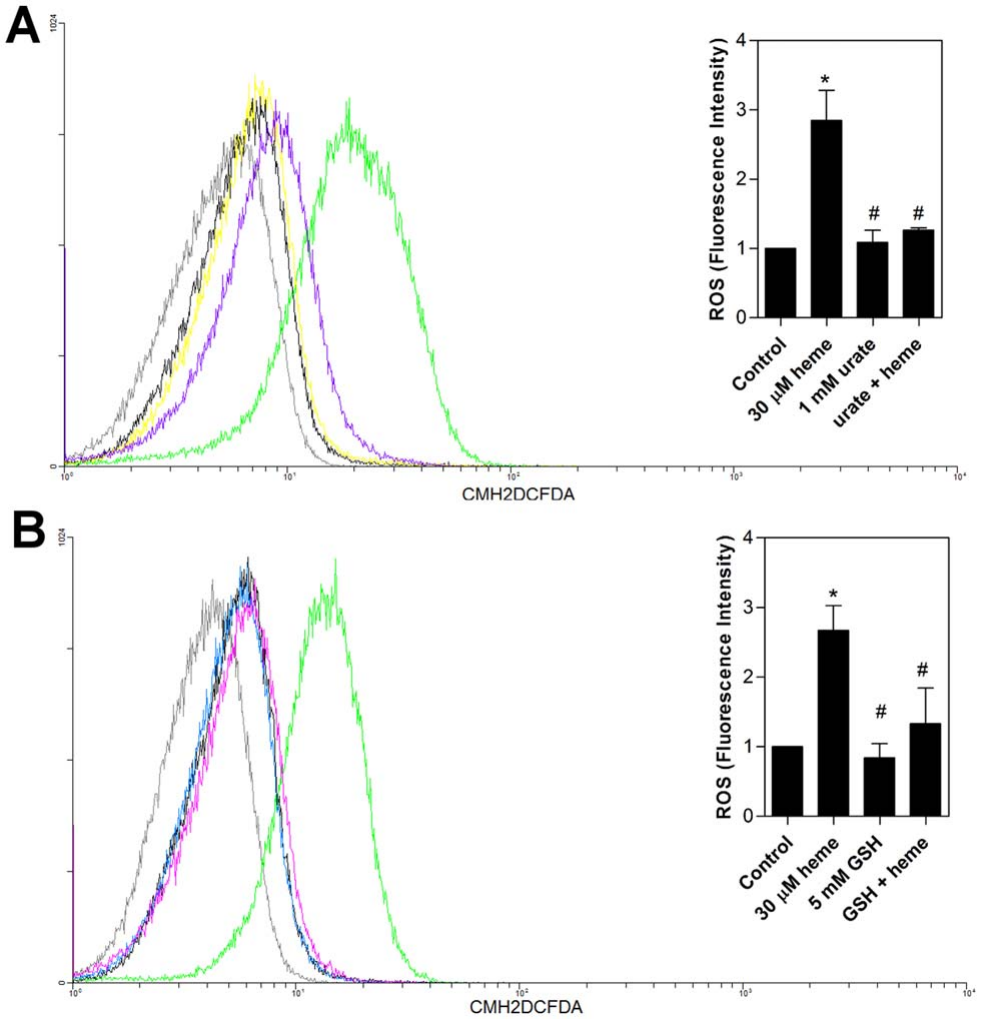
Classical antioxidants are able to prevent ROS induced by heme in *T. cruzi* epimastigotes. (**A**) Epimastigotes (1×10^7^cells/mL) were pre-incubated in PBS containing 2 µM CMH_2_DCFDA for 30 min and treated with 1 mM urate and 30 µM heme for 15 min. ROS formation was analysed by flow cytometry. The histograms show autofluorescence (gray), 2 µM CMH_2_DCFDA (control-black), 30 µM heme (green), 1 mM urate (yellow) and heme+urate (purple). The histograms are representative of four independent experiments. The inset graph shows the fluorescence intensity values obtained by the ratio of the experimental group median to the control group median (without heme). (**B**) Epimastigotes (1×10^7^ cells/mL) were pre-incubated in PBS containing 5 mM GSH for 2 h and incubated with 2 µM CMH_2_DCFDA for 30 min with the addition of 30 µM heme for the final 15 min. The production of ROS was analysed by flow cytometry. The histograms are representative of 4 experiments. The histograms show autofluorescence (gray), 2 µM CMH_2_DCFDA (control-black), 30 µM heme (green), 5 mM GSH (blue) and heme+GSH (pink). The inset graph shows the fluorescence intensity values obtained by the ratio of experimental group median to the control group median (without heme). Data are expressed as the mean ± standard deviation (n = 4), * *p*<0.001 as compared to the control group and ^#^ *p*<0.001 relative to the heme group by Tukey's test.

**Figure 7:**
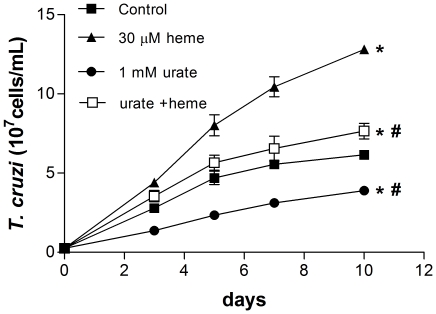
Urate and *T. cruzi* proliferation. Epimastigotes (2.5×10^6^cells/mL) were incubated in BHI medium supplemented with 10% FCS in the absence (control) or in the presence of 30 µM heme and 1 mM urate for ten days. The growth curve is representative of three independent experiments. All data are presented as the mean ± standard deviation (n = 3), * *p*<0.05 compared to the control group or # p<0.05 as compared to the heme group by Tukey's test.

ROS normally occur in living tissues at relatively low steady-state levels because of the large number of antioxidants mechanisms involved in cellular protection. Here, the antioxidants urate (Figure 6A) and GSH (Figure 6B), which are potent ROS scavengers, were able to reverse all fluorescence signals. Assuming that ROS is in fact important for the proliferation of these cells and since antioxidants remove these species from the cell milieu we can suppose that the growth of the parasites does not increase in the presence of antioxidants. So, in order to prove this hypothesis we tested the effect of urate upon the parasite growth. Figure 7 shows that 1 mM urate significantly decreased parasite proliferation, even in the presence of heme, indicating that ROS levels greatly influence the parasite growth homeostasis.

### Heme-induced ROS formation in *T. cruzi* is kinetically regulated

**Figure 8:**
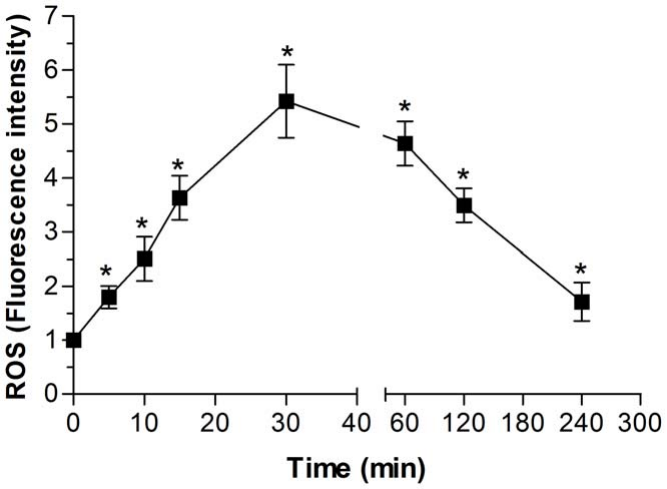
Heme generates ROS in *T. cruzi* in a transient manner. Epimastigotes (1×10^7^cells/mL) were treated in PBS with 30 µM heme for different periods of time (5, 10, 15, 30, 60, 120 and 240 min) and loaded with 2 µM CMH_2_DCFDA in PBS for 30 min. ROS formation was analysed by flow cytometry. Fluoresence intensity values were obtained by the ratio of the heme group median to the control group median. Data are expressed as the mean ± standard deviation (n = 5), * *p*<0.05 as compared to the control group by Tukey's test.

An increased magnitude and longer duration of the ROS concentration demonstrates that the process is not able to protect the cells against ROS. However, a temporary increase of the ROS concentration indicates the presence of a regulatory process that helps the cells or tissues to achieve low, steady-state ROS levels [14]. Next, we investigated the dynamics by which heme induces ROS formation in *T. cruzi*. Figure 8 shows that heme induced a transient increase in ROS formation, as assessed by the fluorescence signal of CMH_2_-DCFDA, and maximum levels of fluorescence were observed after incubation with heme for 30 min. This result shows that in heme-induced ROS formation in *T. cruzi*, there is an important antioxidant mechanism that can efficiently reverse the redox imbalance in a short period. This result suggests the presence of a regulatory event since ROS formation is dependent exclusively on the heme molecule and can be regulated intracellularly very quickly. Changes in the redox balance can indicate the involvement of redox signalling.

### Induction of *T. cruzi* proliferation by heme is mediated by ROS formation and requires CaMKII redox signalling

**Figure 9:**
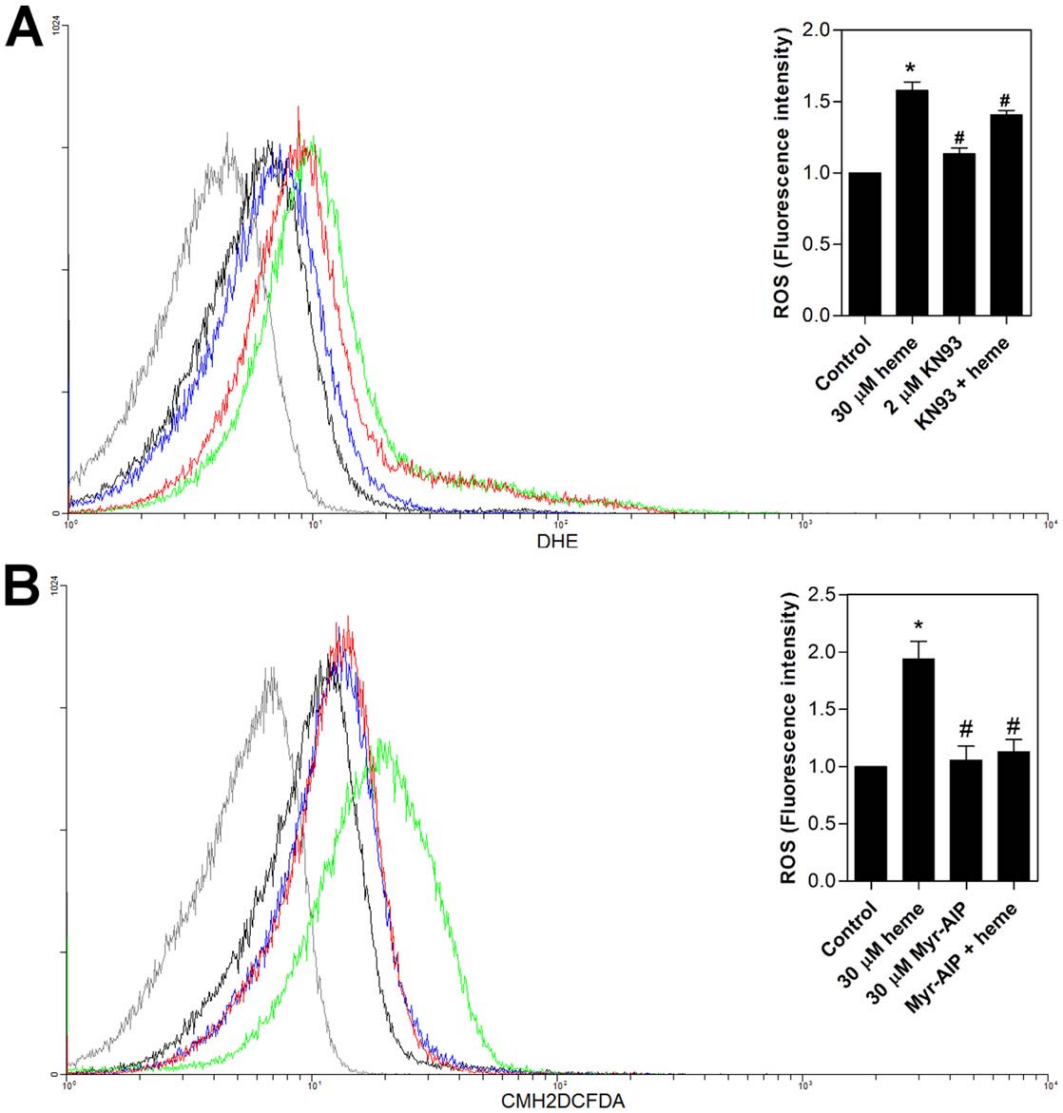
The CaMKII inhibitor is able to prevent ROS formation in *T. cruzi* epimastigotes. (**A**) Epimastigotes (1×10^7^cells/mL) were pre-incubated in PBS with 2 µM KN-93 for 1 h and then incubated in PBS with 5 µM DHE and 30 µM heme for 30 min. The production of ROS was analysed by flow cytometry. The histograms show autofluorescence (gray), 5 µM DHE (control-black), 30 µM heme (green), 2 µM KN-93 (blue) and KN-93+heme (red). The histograms are representative of two independent experiments. The inset graph represents the fluorescence intensity values obtained by the ratio of experimental group median to the control group median (without heme). Data are expressed as the mean ± standard deviation (n = 2), * *p*<0.05 as compared to the control group and ^#^ *p*<0.05 relative to the heme group by Tukey's test. (**B**) Epimastigotes (1×10^7^cells /mL) were pre-incubated in PBS with 30 µM Myr-AIP for 1 h and then loaded with 2 µM CMH_2_DCFDA for 30 min with the addition of 30 µM heme for the final 15 min. ROS formation was analysed by flow cytometry. The histograms show autofluorescence (gray), CMH_2_DCFDA (control- black), 30 µM heme (green), 30 µM Myr-AIP (blue) and Myr-AIP+heme (red). The histograms are representative of three independent experiments. The inset graph represents the fluorescence intensity values obtained by the ratio of the experimental group median to the control group median (without heme). Data are expressed as the mean ± standard deviation (n = 3), * *p*<0.001 as compared to the control group (without heme) and ^#^ *p*<0.001 compared to the heme group by Tukey's test.

In an attempt to elucidate the mechanisms by which heme regulates redox-dependent *T. cruzi* proliferation, we tested the effect of specific inhibitors of several specific enzymes involved in signalling cascades (Table 1). Table 1 shows that heme-induced ROS formation in *T. cruzi* was not affected by protein kinase A (PKA), protein kinase C (PKC), protein kinase G (PKG), cyclin-dependent kinases, or phosphatidyl inositol-3 kinase (PI3K) specific inhibitors. We also tested the CaMKII inhibitors KN-93, a molecule that acts by competitively binding to the calmodulin binding domain of the enzyme and Myr-AIP, a myristoylated form of the specific autocamtide-2-related inhibitory peptide (Figures 9A and 9B). These specific inhibitors of CaMKII, KN-93 and Myr-AIP, were able to block heme-induced ROS formation, strongly implicating the involvement of this enzyme in the redox mechanisms required by heme to promote *T. cruzi* proliferation.

**Table 1:** The effect of kinase inhibitors on ROS production stimulated by heme in *T. cruzi*.

Inhibtor	Target	ROS (Fluorescence intensity)	
		No heme	Heme
Control	----	1	3.240±0.435
H-89 0.24 µM	PKA	1.061±0.085	2.596±0.505
Roscovitine 3.5 µM	cyclin dependent kinase	1.194±0.226	2.643±0.533
BIS 0.05 µM	PKC	1.021±0.017	2.297±0.624
Ly 294002 8 µM	PI3-K	1.468±0.198*	3.305±0.120
H9 5 µM	PKG	1.063±0.016	2.375±0.404

Epimastigotes (1×10^7^cells/mL) were pre-incubated in PBS with the PK inhibitors (5× *Ki*) for 1 h and loaded in PBS with 2 µM CMH_2_DCFDA for 30 min and with 30 µM heme for the final 15 min. ROS formation was measured by flow cytometry. **Fluorescence** intensity values were obtained by the ratio of the experimental group median (with heme) to the control group median (without heme). Data are expressed as the mean ± standard derivation (n = 3),

*p<0.01 as compared to the control group (no heme) by Tukey's test.

**Figure 10:**
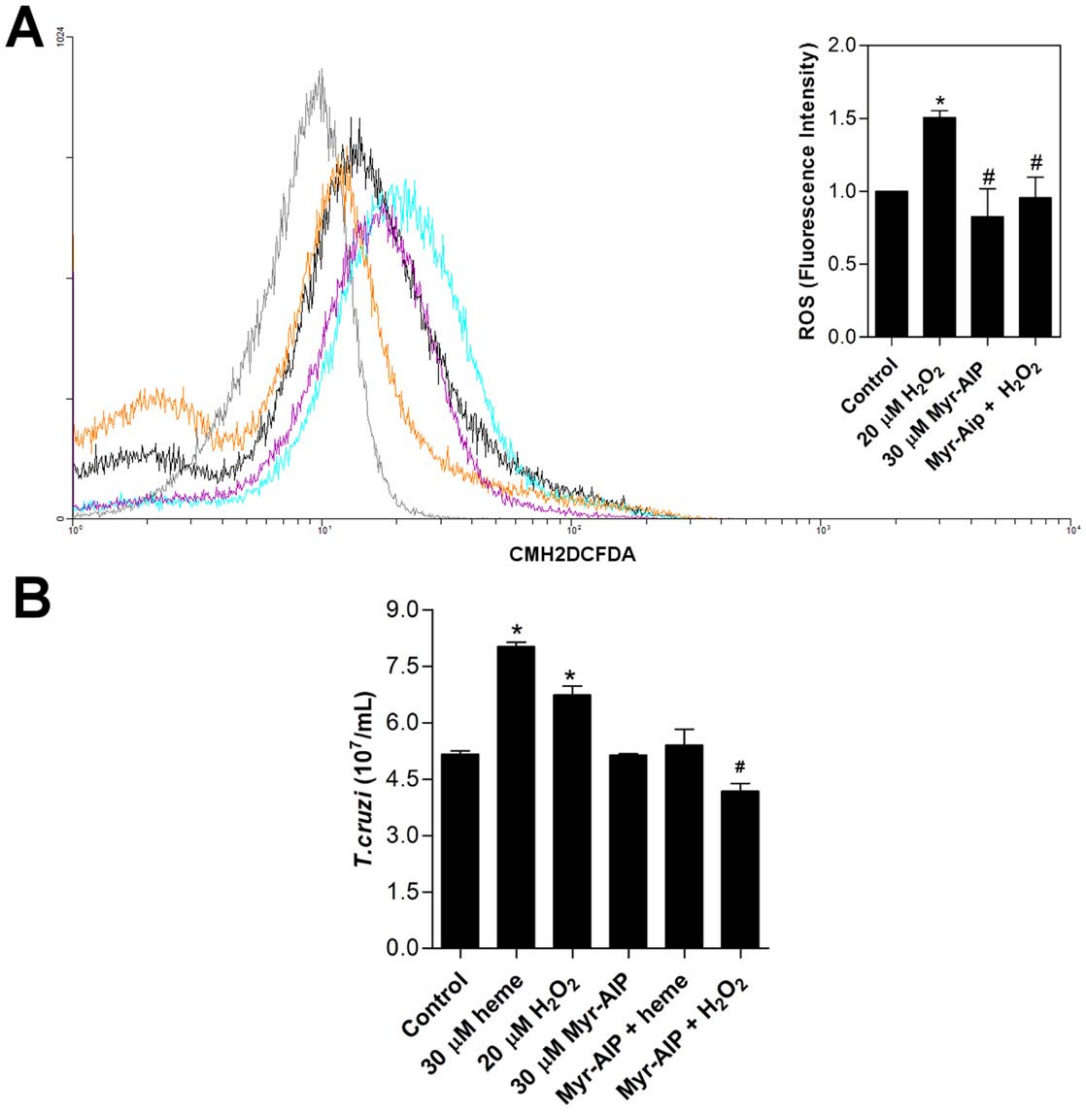
The CaMKII inhibitor is able to prevent ROS formation in *T. cruzi* epimastigotes. (**A**) Epimastigotes (1×10^7^ cells/mL) were pre-incubated in PBS with 30 µM Myr-AIP for 1 h and then incubated in PBS with 2 µM CMH_2_DCFDA and 20 µM H_2_O_2_ for 30 min. The production of ROS was analysed by flow cytometry. The histograms show autofluorescence (gray), 2 µM CMH_2_DCFDA (control-black), 20 µM H_2_O_2_ (blue), 30 µM Myr-AIP (orange) and Myr-AIP+H_2_O_2_ (purple). The histograms are representative of two independent experiments. The inset graph represents the fluorescence intensity values obtained by the ratio of experimental group median to the control group median (without heme). Data are expressed as the mean ± standard deviation (n = 2), * *p*<0.05 as compared to the control group and ^#^ *p*<0.05 relative to the H_2_O_2_ group by Tukey's test. (**B**) Epimastigotes (2.5×10^6^ cells/mL) were incubated in BHI medium supplemented with 10% FCS in the absence (control) or in the presence of 30 µM heme, 20 µMH_2_O_2_, 30 µM Myr-AIP, or 30 µM Myr-AIP plus heme or H_2_O_2_. After five days parasites were quantified using a Neubauer chamber. All data are presented as the mean ± standard deviation (n = 2), * *p*<0.05 as compared to the control group and ^#^ *p*<0.05 relative to the H_2_O_2_ group by Tukey's test.

Next, we determined whether similar to heme, exogenous H_2_O_2_ and pharmacological blockade of CaMKII would exert an inhibitory effect on epimastigotes ROS formation and parasite proliferation. As shown in figure 10A, the specific inhibitor of CaMKII blocked ROS production in epimastigostes. Furthermore, figure 10B shows that the treatment of parasites with Myr-AIP disturbed the H_2_O_2_-induced parasite growth.

In fact, this result is in agreement with previous evidence from our group showing that *T. cruzi* epimastigote proliferation is completely blocked following inhibition of CaMKII activity by Myr-AIP and that subsequent heme-induced proliferation does not occur [31].

## Discussion

Altogether, the data presented herein indicate the importance of the heme molecule, an abundant and important molecule in *Trypanosoma cruzi* biology, and the value of elucidating the defence and regulatory mechanisms developed by this parasite in response to heme. The drugs currently available for the treatment of Chagas disease (benznidazole , nifurtimox) seem to act by inducing oxidative stress [36], [37]. In this regard, the identification of systems involved in the formation and detoxification of ROS as well as its role in the life cycle of the parasite, provide a valuable target for the development of an effective chemotherapy. The data presented herein demonstrate that heme is able to induce ROS in a dose-dependent manner in *T. cruzi* epimastigotes favouring the increase of the parasite proliferation. Also, the impairment of the parasite proliferation by the antioxidant urate, corroborate the idea that redox signalling in fact governs *T. cruzi* biology. Although the heme molecule serves as a component of many essential enzymatic activities, as an amphiphilic compound, it may promote deleterious cellular processes such as lipid peroxidation and oxidative membrane damage [8], [11], [33]. Consistent evidence indicates that heme-induced lipid peroxidation is exerted mainly via the decomposition of organic hydroperoxides – instead of H_2_O_2_ – into alkoxyl and peroxyl radicals [38], [39]. The measurement of TBARS revealed that heme induced lipid peroxidation after 30 min of incubation in a dose-dependent manner, corroborating our results demonstrating a peak of ROS formation after 30 min. In the present study, immunoblotting against 4-HNE adducts indicated an increase of 4-HNE-modified proteins in parasites exposed to heme as compared to unexposed parasites. These results suggest that *T. cruzi* might benefit from the induction of aldehydes with biological activities necessary for the modulation of its cellular signalling. In fact, recent reports indicate that 4-HNE is a potent cell signalling molecule [26], [34]. The present findings corroborate the data in the literature demonstrating that 4-HNE is involved in the proliferation and differentiation of several cell types *in vitro* [35].

Current perspectives favour evidence for the existence of a redox-based network of regulatory mechanisms that are intimately linked to cellular function. Unlike oxidative stress, which is characterised by an increase of ROS and radical-induced damage, redox regulation, or “redox signalling,” describes a reversible phase of physiological regulatory reactions that occur over shorter time periods. In such processes, the oxidative reactions are returned to the resting state through the activation of reductive pathways [15], [17], [22], [40], [41]. PI3K, MAPK and CaMKs have been related to redox signalling. ROS-induced kinases activation appears to occur, at least in part, through the inactivation of phosphatases, which can occur via the oxidation of these proteins [42]–[46]. These observations lead us to suggest that heme could be involved in ROS formation in *T. cruzi* epimastigotes by modulating an intracellular signalling pathway that is redox-sensitive.

Among all of the pathways tested, inhibition of the PI3K increased ROS formation by approximately 47% independently of the presence of heme. Goldshmit *et al.* [47] suggested that the PI3K pathway regulates the toxic levels of ROS induced by oxidative stress in neurons. Recent studies have shown that CaMKs can function as sensors of the redox status of different cellular types. Oxidative stress induced by H_2_O_2_ activates CaMKII and CaMKIV in T Jurkat lymphocytes independently of calcium influx in these cells [29], [40], thus stimulating the antiapoptotic pathway through IkB and Akt [29]. These events are probably due to the oxidation and inactivation of intracellular phosphatases.

Recently, Cosentino-Gomes *et al* have shown that hydrogen peroxide inhibits ecto-phosphatase in *Trypanosoma rangeli* [48]. According to these observations, any cellular process that involves ROS production could potentially activate CaMKs, even in the absence of a calcium influx. Through pharmacological and molecular inhibition, it has been demonstrated that CaMKII participates in ERK phosphorylation induced by H_2_O_2_ in human breast cancer cells [49]. This pathway could be one of the pathways responsible for allowing cancer cells to survive treatments that induce oxidative stress, such as chemotherapy or ionising radiation [49]. In addition, Bouallegue *et al*, demonstrated that CaMKII act as a critical upstream component triggering the H_2_O_2_-induced phosphorylation of IGF-1R, ERK and PKB in vascular smooth muscle cells [50]. In this case, the ROS induced upregulation of CaMKII could contribute to the abnormal cell proliferation related to the pathogenesis of vascular disease [50], [51].

We have searched the *Trypanosoma cruzi* genome for CaMKII homologs and identified two ORFs that encode putative CaMKII in the database representing two alleles of the same gene: one allele sequenced from strain CL Brenner, non-Esmeraldo-like haplotype (XP_815126) and another allele sequenced from strain CL Brenner, Esmeraldo-like haplotype (XP_816286) (Figure S1). These two alleles are extremely similar among themselves (7 different residues in a total of 545) and both present approximately 37% identity and 55% similarity to the kinase domain (residues 23–271) of the human CaMKII alpha subunit [52]. These sequences had been previously identified as members of the CaMK family by Parsons and coworkers [53]. We have used in this and previous work [31] reagents considered to be specific for the identification of CaMKII protein activation (phospho-specific CaMKII antibody) and activity (the substrate camtide-2 and the inhibitor Myr-AIP). All three reagents were developed based on the aminoacid sequence neighbouring the autophosphorylation T^286^ residue from CaMKII subunits. In Figure S2 we show that this region is conserved in the *T. cruzi* sequences identified giving support to the use of these reagents to study the function of CaMKII in *T. cruzi* [52]. The results of these studies support the hypothesis that a CaMKII-like enzyme is involved in the redox imbalance, thus modulating the adaptation to the redox status in different cell types such as *Trypanosoma cruzi*. In this present work we demonstrate that, despite the presence of large amounts of heme within the insect midgut, which is assumed to cause redox imbalance to the insect [2], [54], *T. cruzi* epimastigotes require heme for proliferation in a mechanism that involves parasite CaM kinase II-like activation. Additionally, this is another indication for the beneficial effect of ROS if is tightly controlled.

Furthermore, these two *T. cruzi* sequences presents a conserved calmodulin binding region (residues 290–300 of the human CaMKII alpha subunit – Figure S1) and an autoinhibitory domain containing a threonine residue in a position similar to the T286 residue of the human CaMKII alpha subunit (marked in green in Figure S1). The autophosphorylation of the T286 residue is directly involved in the regulation of a calcium/calmodulin independent activity that is observed after the first activation of this enzyme by calcium/calmodulin binding [49]. This characteristic distinguishes CaMKII from other members of the calcium/calmodulin dependent protein kinase family [49]. Another feature of CaMKII enzyme that differ them from other members of the CaM-dependent protein kinase family is the formation of a dodecamer complex. This association occurs via its C-terminal region [49] and although the similarity is not as good as in other functional domains, we can observe in the alignment (Figure S1) [52] an overall conservation of the characteristics in this region as well. Therefore, the conservation of these features (kinase domain, calcium/calmoduling binding region, autophosphorylation site and C-terminal association domain) on these two *T. cruzi* sequences makes a strong argument for their election as putative CaMKII enzymes.

The biochemical interplay between *T. cruzi* and the triatomine vectors has been investigated since 1909 [1]. Notwithstanding, a comprehensive study describing the physiological role of heme in *T. cruzi*-vector interaction has been lacking. Several important issues must be considered in this context: *i*) the total heme levels in the vector midgut reach millimolar concentrations; of note, heme crystallisation into hemozoin (Hz) is a very efficient heme detoxification process that takes place in the midgut of different triatomine species [55], [56]. Interestingly, recent evidence has demonstrated that Hz represents by far the dominant iron-containing compound found in the triatomine midgut, comprising at least 97% of whole iron species [57]. Therefore, despite the very low “*free*” heme levels in the *R. prolixus* midgut [57], it is conceivable that small amounts of heme would be physiologically relevant to allow progression of the *T. cruzi* life cycle in this compartment. *ii*) at micromolar concentrations, heme exerts a potent pro-oxidant effect, *iii*) the drugs currently available for the treatment of Chagas disease (nifurtimox and benznidazole) seem to act via the alteration of redox metabolism [36], [37], and novel drug candidates, such as the naphthofuranquinones and the putrescine analogue diaminobutanone, exert their trypanocidal activity by causing a mitochondrial dysfunction that results in increased ROS generation [58], [59]. Thus, the identification of mechanisms involved in ROS formation and detoxification as well as their role in the *T. cruzi* life cycle would provide valuable data for the development of novel, effective therapeutic approaches.

The heme molecule represents a key molecule in the interface between the vector and the parasite and this interaction determine the transmition of Chagas disease. In this study we demonstrate that ROS (H_2_O_2_) or heme-induced ROS activated CaMKII, triggering the proliferation of the epimatigote forms. Also, the antioxidants, such as urate and GSH, inhibited heme-induced ROS and parasite proliferation. In addition, Myr-AIP, the specific CaMKII inhibitor extinguished heme-induced ROS in epimastigotes, decreasing parasite growth. The data presented herein indicate that heme induces a transient oxidative stress condition that stimulates *T. cruzi* proliferation via a mechanism mediated by a CaM Kinase II-like pathway.

## Supporting Information

Figure S1 **Multiple sequence alignment of human CaMKII isoforms and two putative isoforms of CaMKII from** ***T. cruzi*** **.** Asterisk “*” means that the residues are identical in all sequences in the alignment. “**:**” means that conserved substitutions have been observed, while “**.**” means that semi-conserved substitutions are observed. Residues marked in red are the 7 different aminoacids observed between the two *T. cruzi* isoforms. Residues marked in green are homologous to the T^286^ from the human CaMKII alpha isoform which is phosphorylated during the process of autophosphorylation/ autoactivation induced by calcium/calmodulin binding. The alignment was made using CLUSTALW program version 2.0.12 (52).
(DOC)

Figure S2 **Sequence alignment of Myr-AIP with human and** ***T. cruzi*** **isoforms of CaMKII.** Alignment of the aminoacid sequences of MyrAIP (**A**), the peptide epitope used to generate the phospho-CaMKII antibody (**B**) and Camtide2 (**C**) with the homologous region of human and *T. cruzi* CamKII isoforms. XP_816286 sequence was omitted from the figure since it is identical to the sequence of XP_815126. Residue highlighted in yellow indicates the T286A mutation introduced in Myr-AIP sequence to inhibit the phosphorylation of this peptide. Asterisk “*” means that the residues are identical in all sequences in the alignment. “**:**” means that conserved substitutions have been observed, while “**.**” means that semi-conserved substitutions are observed.
(DOC)
