# Supplementary material for: Heme-Induced ROS in Trypanosoma Cruzi Activates CaMKII-Like That Triggers Epimastigote Proliferation. One Helpful Effect of ROS
Source: PLoS One. 2011 Oct 11;6(10):e25935. doi: 10.1371/journal.pone.0025935 (PMC3191175; doi:10.1371/journal.pone.0025935)
Supplement: Figure S1 — Multiple sequence alignment of human CaMKII isoforms and two putative isoforms of CaMKII from T. cruzi . Asterisk “*” means that the residues are identical in all sequences in the alignment. “:” means that conserved substitutions have been observed, while “.” means that semi-conserved substitutions are observed. Residues marked in red are the 7 different aminoacids observed between the two T. cruzi isoforms. Residues marked in green are homologous to the T286 from the human CaMKII alpha isoform which is phosphorylated during the process of autophosphorylation/ autoactivation induced by calcium/calmodulin binding. The alignment was made using CLUSTALW program version 2.0.12 (52). (DOC) [file pone.0025935.s001.doc]

CaMKII_gamma -------MATTATCT--------RFTDDYQLFEELGKGAFSVVRRCVKKTS--TQEYAAK 43

CaMKII_delta -------MASTTTCT--------RFTDEYQLFEELGKGAFSVVRRCMKIPT--GQEYAAK 43

CaMKII_beta -------MATTVTCT--------RFTDEYQLYEDIGKGAFSVVRRCVKLCT--GHEYAAK 43

CaMKII_alpha --------MATITCT--------RFTEEYQLFEELGKGAFSVVRRCVKVLA--GQEYAAK 42

XP_815126 MDFQALQSTLTEVCKGTSLKRGVSFDQRYELEVEIGKGAYGTVWRCHRRFDSMRRPYGVK 60

XP_816286 MDFQALQSTLTEVCKGTSLKRGVSFDQRYELEAEIGKGAYGTVWRCHRRFDSMRRPYGVK 60

* .*. * : *:* ::****:..* ** : : *..*

CaMKII_gamma IINTKKLSARDHQKLEREARICRLLKHPNIVRLHDSISEEGFHYLVFDLVTGGELFEDIV 103

CaMKII_delta IINTKKLSARDHQKLEREARICRLLKHPNIVRLHDSISEEGFHYLVFDLVTGGELFEDIV 103

CaMKII_beta IINTKKLSARDHQKLEREARICRLLKHSNIVRLHDSISEEGFHYLVFDLVTGGELFEDIV 103

CaMKII_alpha IINTKKLSARDHQKLEREARICRLLKHPNIVRLHDSISEEGHHYLIFDLVTGGELFEDIV 102

XP_815126 IINKKKAGAKGLKWVMGEVETMSLLIHPNIVRLEETFQDEENLWIVMEYMPGGELRSAVL 120

XP_816286 IINKKKAGAKGLKWVMGEVETMSLLIHPNIVRLEETFQDEENLWIVMEYMPGGELRSAVL 120

***.** .*:. : : *.. ** *.*****.:::.:* ::::: :.**** . ::

CaMKII_gamma AREYYSEADASHCIHQILESVNHIHQHDIVHRDLKPENLLLASKCKGAAVKLADFGLAIE 163

CaMKII_delta AREYYSEADASHCIQQILESVNHCHLNGIVHRDLKPENLLLASKSKGAAVKLADFGLAIE 163

CaMKII_beta AREYYSEADASHCIQQILEAVLHCHQMGVVHRDLKPENLLLASKCKGAAVKLADFGLAIE 163

CaMKII_alpha AREYYSEADASHCIQQILEAVLHCHQMGVVHRDLKPENLLLASKLKGAAVKLADFGLAIE 162

XP_815126 RDGIFSEAQARRITTQLLLALEFIHQNGIVHRDMKPENCLLSEG--DLVCKISDFGFSVL 178

XP_816286 RDGIFSEAQARRITTQLLLALEFIHQNGIVHRDMKPENCLLSEG--DLVCKISDFGFSVL 178

:***:* : *:* :: . * .:****:**** **:. . . *::***:::

CaMKII_gamma VQGEQQAWFGFAGTPGYLSPEVLRKDPYGKPVDIWACGVILYILLVGYPPFWDEDQHKLY 223

CaMKII_delta VQGDQQAWFGFAGTPGYLSPEVLRKDPYGKPVDMWACGVILYILLVGYPPFWDEDQHRLY 223

CaMKII_beta VQGDQQAWFGFAGTPGYLSPEVLRKEAYGKPVDIWACGVILYILLVGYPPFWDEDQHKLY 223

CaMKII_alpha VEGEQQAWFGFAGTPGYLSPEVLRKDPYGKPVDLWACGVILYILLVGYPPFWDEDQHRLY 222

XP_815126 VGSDQ-CLMSFCGTTVFMAPEIFGDTSYGKPVDMWAIGVMVYFMVTGTYPFTGRSHRELT 237

XP_816286 VGSDQ-CLMSFCGTTVFMAPEIFGDTSYGKPVDMWAIGVMVYFMVTGTYPFTGRSHRELT 237

* .:* . :.*.**. :::**:: . .******:** **::*:::.* ** ...::.*

CaMKII_gamma QQIKAGAYDFPSPEWDTVTPEAKNLINQMLTINPAKRITADQALKHPWVCQRSTVASMMH 283

CaMKII_delta QQIKAGAYDFPSPEWDTVTPEAKDLINKMLTINPAKRITASEALKHPWICQRSTVASMMH 283

CaMKII_beta QQIKAGAYDFPSPEWDTVTPEAKNLINQMLTINPAKRITAHEALKHPWVCQRSTVASMMH 283

CaMKII_alpha QQIKAGAYDFPSPEWDTVTPEAKDLINKMLTINPSKRITAAEALKHPWISHRSTVASCMH 282

XP_815126 DAICGGRCNLKSGRIAEGSASLRDFISMLLVVDPNRRLSAREALKHPWIKLGMNMGNSIR 297

XP_816286 DAICGGRCNLKSGRIAEGSASLRDFISMLLVVDPNRRLSAREALKHPWIKLGMNMGNSIR 297

: * .* :: * . :.. :::*. :*.::* :*::* :******: .:.. ::

CaMKII_gamma RQETVECLRKFNARRKLKGAILTTMLVSR-----NFSA---------------------- 316

CaMKII_delta RQETVDCLKKFNARRKLKGAILTTMLATR-----NFSA---------------------- 316

CaMKII_beta RQETVECLKKFNARRKLKGAILTTMLATR-----NFSVGRQTTAPATMSTAASGTTMGLV 338

CaMKII_alpha RQETVDCLKKFNARRKLKGAILTTMLATR-----NFSG---------------------- 315

XP_815126 -DETELKRHGLRPRSIFRAAIIALMAAHRLCYLR-------------------------- 330

XP_816286 -DETELKRHGLRPRSIFRAAIIALMAAHRLCYLR-------------------------- 330

:** : :..* ::.**:: * . *

CaMKII_gamma --AKSLLNKKSDGGVKPQSNNKNSLVSPAQEPAPLQTAMEPQTTVVHNATDGIKGSTESC 374

CaMKII_delta --AKSLLKK----------------------------------------PDGVKESTESS 334

CaMKII_beta EQAKSLLNKKADGVKPQTNSTKNSAAATSPKGTLPPAALEPQTTVIHNPVDGIKESSDSA 398

CaMKII_alpha --GKSGGNKKSDGVKKRKSSS----------------------------SVQLMESSEST 345

XP_815126 -YCRMLENNFCSAFTILRNFRFAVSGAYEPPCPTLDCSG---VFAKHPRGVRFLLPMLEV 386

XP_816286 -YCRMLENNFCSAFTILRNFRFAVSGAYEPPSPTLDCSG---VFARHPRGVRFLLPMLEV 386

: :: . . .

CaMKII_gamma NTTTEDEDLKAAPL---------------------------------------------- 388

CaMKII_delta NTTIEDEDVKAR------------------------------------------------ 346

CaMKII_beta NTTIEDEDAKAPRVPDILSSVRRGSGAPEAEGPLPCPSPAPFSPLPAPSPRISDILNSVR 458

CaMKII_alpha NTTIEDEDTKVR------------------------------------------------ 357

XP_815126 SRTIEALDLSSNNIDN-------------------------------------------- 402

XP_816286 SRTIEALDLSSNNIDN-------------------------------------------- 402

. * * * .

CaMKII_gamma ----------------------------------------RTGNGSSVPEGRSSRDRTAP 408

CaMKII_delta ------------------------------------------------------------

CaMKII_beta RGSGTPEAEGPLSAGPPPCLSPALLGPLSSPSPRISDILNSVRRGSGTPEAEGPSPVGPP 518

CaMKII_alpha ------------------------------------------------------------

XP_815126 ------------------------------------------------------------

XP_816286 ------------------------------------------------------------

CaMKII_gamma SAGMQPQPSLCSSAMRKQEIIKITEQLIEAINNGDFEAYTKICDPGLTSFEPEALGNLVE 468

CaMKII_delta ----------------KQEIIKVTEQLIEAINNGDFEAYTKICDPGLTAFEPEALGNLVE 390

CaMKII_beta PCPSPTIPGPLPTPSRKQEIIKTTEQLIEAVNNGDFEAYAKICDPGLTSFEPEALGNLVE 578

CaMKII_alpha ----------------KQEIIKVTEQLIEAISNGDFESYTKMCDPGMTAFEPEALGNLVE 401

XP_815126 ------------LTLFQQLAKTVGQHPTLVSLNLSFNPIPLLAGRGLLRLARSPQSKLLH 450

XP_816286 ------------LTLFQQLAKTVGQHPTLVSLNLSFNPIPLLASRGLLRLARSPQSKLLH 450

:* . :: . * .*:. . :.. *: : .. .:*:.

CaMKII_gamma G-MDFHKFYFENLLSKNSKPIHTTILNPHVHVIGEDAACIAYIRLTQYIDGQGRPRTSQS 527

CaMKII_delta G-MDFHRFYFENALSKSNKPIHTIILNPHVHLVGDDAACIAYIRLTQYMDGSGMPKTMQS 449

CaMKII_beta G-MDFHRFYFENLLAKNSKPIHTTILNPHVHVIGEDAACIAYIRLTQYIDGQGRPRTSQS 637

CaMKII_alpha G-LDFHRFYFENLWSRNSKPVHTTILNPHIHLMGDESACIAYIRITQYLDAGGIPRTAQS 460

XP_815126 LGLDGTLLPSETIAQISAALKEKAVVSSAAAFTPQLTPATRSLEISTSLSRNNLSVSSPR 510

XP_816286 LGLDGTLLPSETIAQISAALKEKAVVSSAAAFTPQLTPATRSLEISTSLSRNNLSVSSPR 510

:* : *. . .. ::.. . : :.. :.:: :. . . :

CaMKII_gamma EETRVWHRRDGKWLNVHYHCSGAPAAPLQ------ 556

CaMKII_delta EETRVWHRRDGKWQNVHFHRSGSPTVPIK------ 478

CaMKII_beta EETRVWHRRDGKWQNVHFHCSGAPVAPLQ------ 666

CaMKII_alpha EETRVWHRRDGKWQIVHFHRSGAPSVLPH------ 489

XP_815126 QRFGVLHSPFTRQPVRKTTEPRLPPLSNTKRRAGK 545

XP_816286 QRFVVPHSPFTRQPVRKTTEPRLPPLSNTRRRAGK 545

:. * * : : . *

Supplementary figure 1
