# Supplementary material for: Heme-Induced ROS in Trypanosoma Cruzi Activates CaMKII-Like That Triggers Epimastigote Proliferation. One Helpful Effect of ROS
Source: PLoS One. 2011 Oct 11;6(10):e25935. doi: 10.1371/journal.pone.0025935 (PMC3191175; doi:10.1371/journal.pone.0025935)
Supplement: Figure S2 — Sequence alignment of Myr-AIP with human and T. cruzi isoforms of CaMKII. Alignment of the aminoacid sequences of MyrAIP (A), the peptide epitope used to generate the phospho-CaMKII antibody (B) and Camtide2 (C) with the homologous region of human and T. cruzi CamKII isoforms. XP_816286 sequence was omitted from the figure since it is identical to the sequence of XP_815126. Residue highlighted in yellow indicates the T286A mutation introduced in Myr-AIP sequence to inhibit the phosphorylation of this peptide. Asterisk “*” means that the residues are identical in all sequences in the alignment. “:” means that conserved substitutions have been observed, while “.” means that semi-conserved substitutions are observed. (DOC) [file pone.0025935.s002.doc]

**A**

**CaMKII_alp** TVASCMHRQETVDCLKK...

**CaMKII_bet** TVASMMHRQETVECLKK...

**CaMKII_gam** TVASMMHRQETVECLRK...

**CaMKII_del** TVASMMHRQETVDCLKK...

**XP_815126**  NMGNSI-RDET-E-LKR...

**Myr_AIP**  --KKALRRQEAVDAL--...

. : *:*: : *

**B**

**CaMKII_alp** TVASCMHRQEpTVDCLKK...

**CaMKII_bet** TVASMMHRQEpTVECLKK...

**CaMKII_gam** TVASMMHRQEpTVECLRK...

**CaMKII_del** TVASMMHRQEpTVDCLKK...

**XP_815126**  NMGNSI-RDEpT-E-LKR...

**Ab_epitope** ----KMHRQEpTVDCLK-...

: *:* * : **

**C**

**CaMKII_alp** TVASCMHRQETVDCLKK...

**CaMKII_bet** TVASMMHRQETVECLKK...

**CaMKII_gam** TVASMMHRQETVECLRK...

**CaMKII_del** TVASMMHRQETVDCLKK...

**XP_815126**  NMGNSI-RDET-E-LKR...

**Camtide2**  --KKALRRQETVDAL--...

. : *:** : *

Supplementary figure 2
